# Design considerations in a sib-pair study of linkage for susceptibility loci in cancer

**DOI:** 10.1186/1471-2350-9-64

**Published:** 2008-07-10

**Authors:** Richard A Kerber, Christopher I Amos, Beow Y Yeap, Dianne M Finkelstein, Duncan C Thomas

**Affiliations:** 1 Population Sciences Program, Hunstman Cancer Institute, Salt Lake City, UT, USA; 2 Department of Epidemiology, M. D. Anderson Cancer Center, Houston, TX, USA; 3 Biostatistics Center, Massachusetts General Hospital, Boston, MA, USA; 4 Department of Preventive Medicine, University of Southern California, Los Angeles, CA, USA

## Abstract

**Background:**

Modern approaches to identifying new genes associated with disease allow very fine analysis of associaton and can be performed in population based case-control studies. However, the sibpair design is still valuable because it requires few assumptions other than acceptably high penetrance to identify genetic loci.

**Methods:**

We conducted simulation studies to assess the impact of design factors on relative efficiency for a linkage study of colorectal cancer. We considered two test statistics, one comparing the mean IBD probability in affected pairs to its null value of 0.5, and one comparing the mean IBD probabilities between affected and discordant pairs. We varied numbers of parents available, numbers of affected and unaffected siblings, reconstructing the genotype of an unavailable affected sibling by a spouse and offspring, and elimination of sibships where the proband carries a mutation at another locus.

**Results:**

Power and efficiency were most affected by the number of affected sibs, the number of sib pairs genotyped, and the risk attributable to linked and unlinked loci. Genotyping unaffected siblings added little power for low penetrance models, but improved validity of tests when there was genetic heterogeneity and for multipoint testing. The efficiency of the concordant-only test was nearly always better than the concordant-discordant test. Replacement of an unavailable affected sibling by a spouse and offspring recovered some linkage information, particularly if several offspring were available. In multipoint analysis, the concordant-only test was showed a small anticonservative bias at 5 cM, while the multipoint concordant-discordant test was generally the most powerful test, and was not biased away from the null at 5 cM.

**Conclusion:**

Genotyping parents and unaffected siblings is useful for detecting genotyping errors and if allele frequencies are uncertain. If adequate allele frequency data are available, we suggest a single-point affecteds-only analysis for an initial scan, followed by a multipoint analysis of affected and unaffected members of all available sibships with additional markers around initial hits.

## Background

The sib pair design has been widely used in human studies for mapping genes that affect both quantitative and dichotomous traits. The objective of sibling studies is to determine whether the siblings (or other close relatives) tend to express the same disease phenotype (or similar values of a quantitative trait) when they share a commonly inherited genomic segment measured by genetic markers. Because two loci located close together on the same chromosome tend to be inherited together, sibs who have inherited an allele predisposing them to disease will also inherit a variety of other genetic characteristics located in the same genomic segment as the gene responsible for disease susceptibility. By examining, in a set of pairs of affected sibs, the degree of sharing of genetic marker alleles located throughout the genome, researchers can identify regions that are shared more often than would be expected by chance. This excess allele sharing constitutes the statistical evidence for linkage between the shared markers and putative disease susceptibility loci.

The popularity of the sib-pair and related small-family study designs reflects several attractive characteristics. For highly penetrant alleles, extended families can often be recruited and so provide a powerful source of information for disease localization and fine mapping. However, for complex diseases that result from interactions among loci or that result from both genetic and environmental effects, large pedigrees that include multiple affected individuals may be rare and may not reflect a single etiologically-related genetic factor. Since the pioneering work of Haseman and Elston [1] on quantitative trait mapping, many alternative approaches and extensions to the design have been developed to handle multilocus data [2], extended relative pairs [3-6], consideration of environmental risk factors [7-9], and other situations encountered in studies of complex diseases. In concept, for the study of complex diseases, the design and execution of a sib-pair study is simple. Straightforward power calculations (e.g. [10]) suggest that adequate power to detect linkage might be obtained with relatively modest sample sizes even for complex, low-penetrance susceptibility syndromes. Readily available software packages [11] obtain exact IBD sharing distributions and compute linkage tests using exact methods without requiring explicit specification of an inheritance model, penetrance function, or disease allele frequencies. Numerous authors have observed that nonparametric or model-free linkage statistics, in spite of their name, make hidden assumptions about mode of inheritance and penetrance (e.g., [12-14]) which determine the situations in which they become most powerful. Nevertheless, these methods have been highly popular because of the ease in implementing them and in interpreting the results, as well as the perception that they remain powerful across a broad spectrum of underlying genetic mechanisms.

The modern approaches to identifying new genes associated with disease allow very fine analysis of associaton and can be performed in population based case-control studies. However, the sibpair design is still valuable because it requires few assumptions other than acceptably high penetrance to identify genetic loci. There remains skepticism about relying solely on genome-wide-association studies [15], and sib-pair studies continue to be utilized and published in the literature [16-18]. While association based studies have provided many novel insights into the genetic architecture of complex diseases, they require that linkage disequilibrium is present between genetic markers and disease causing variants. Such a requirement might not be met for diseases that are due to susceptibility loci undergoing recurrent mutations [19] or for genomic regions that show copy number variation and so do not yield reliable SNP data without the implementation of specialized procedures because of deviations from Hardy-Weinberg disequilibrium [20].

Sibships and small families are relatively easy to identify and recruit (compared with designs that make use of large multiplex families). Moreover, a number of familial disease registries and high-risk clinics have been established and have enrolled substantial numbers of sib pairs and other groupings of affected relatives, so that in many cases recruitment has already occurred. In practice, however, the design of an effective small-family linkage study requires more care than is immediately apparent, particularly for complex diseases such as cancer, in which late onset, incomplete penetrance, large numbers of phenocopies, gene-environment interaction, and locus heterogeneity must be anticipated. The purpose of this paper is to evaluate the impact of choices of family sampling strategies in selecting the design of a study aimed at identifying novel genetic factors that predispose individuals to colon cancer. The study is a collaborative effort by two National Cancer Institute-sponsored consortia investigating the genetics of cancer: the Cancer Genetics Network (CGN) and Cooperative Family Registry for Colon Cancer Studies (CFRCCS).

All population based studies of colon cancer have documented its strong familiality [21-24]. Historical cohort studies show an approximately 2.5 fold increased risk for colorectal cancer in the first degree relatives of colorectal cancer cases compared to first degree relatives of unaffected controls [25-28]. Nongenetic factors specifically implicated as protecting individuals from risk for colon cancer include use of nonsteroidal anti-inflammatory drugs, a diet rich in leafy vegetables, and exercise [29-34]. Although these environmental risk factors have been identified as substantially affecting the risk for colon cancer they display only a weak correlation in family members, which is insufficient to explain the high familial risk [35]. Moreover, Khoury, et al. [36] point out that both the correlations of exposure among siblings and relative risks due to shared exposures need to be very large in order to produce observable increases in relative risk to siblings of cases. It seems unlikely that such a combination of highly correlated exposures and large relative risk exists for colorectal cancer.

## Methods

The choice of sampling schemes for a colon sibpair study requires evaluation, as several different options exist simultaneously for the sampling plan (how many families and which relatives to sample), disease model (penetrance, allele frequency, etc), and statistical test of linkage. These aspects, in addition to the method of generating the data sets for the simulations comprise the methods of determining an optimal design strategy for a colon sibpair study. In our analysis of design issues we have pursued a deterministic simulation approach. The first step was to check that the tests we considered had appropriate size, using extensive simulations. For those tests that were valid, we then performed a moderate number of simulations of a large number of families, to obtain the noncentrality parameters describing the behavior of each design configuration and test. We were then able to compare the attributes of these studies across the different design configurations genetic models and tests by fitting regression models, as further described below.

### Disease Model Parameter Choices

#### Genetic Heterogeneity

The genetic basis of many complex human diseases, including some common cancers, is known to include mutations in loci that confer a high penetrance to carriers. In order to minimize the impact that such genetic heterogeneity may have on the statistical efficiency to identify novel loci, gene-hunting studies are often restricted to those cases that have been determined not to be related to any known susceptibility locus. For example, the second susceptibility locus for breast cancer, *BRCA2*, was mapped by Wooster *et al*. [37] based on high-risk families for whom *BRCA1 *linkage was excluded. Genetic syndromes such as juvenile polyposis, Peutz-Jeghers syndrome and familial adenomatous polyposis can be readily eliminated prior to sample collection on the basis of phenotypic characteristics. However, mutations in mismatch repair (MMR) loci such as hMSH2 or hMLH1 cannot be excluded without performing molecular studies. (Measures of tumor microsatellite instability or immunohistochemistry can be helpful in identifying likely carriers, but are neither fully sensitive nor specific.) Estimating the proportion of affected families that might be excluded from analysis because they carry MMR mutations is difficult. Population-based estimates of the prevalence of germ-line MMR mutations among subjects diagnosed with colorectal cancer vary widely even within populations of European descent [38-41], and only a few estimates are available for other ethnic groups [42,43]. Moreover, recruiting from high-risk clinics may lead to disproportionate over-representation of families who have MMR mutations. One large study in Finland [44] found that 12% of affected sib pairs (ASP) showed MMR defects.

The basic assumption for our studies was thus a two-locus non-interacting model, with a rare high-penetrance gene (e.g. *hMSH2/hMLH1 *for HNPCC in the case of colon cancer), unlinked to the search region for new loci and a more common but less penetrant gene being sought by the linkage analysis. We also evaluated the effect of experimental elimination of a previously-known predisposition syndrome such as HNPCC. We investigated both the power and the relative efficiency of different designs under a range of assumptions about the contribution of an additional unlinked locus. The single unlinked locus with varying allele frequency and penetrance that we simulated could also approximate the effects of a few unlinked noninteracting alleles. Although our efforts focused on a study of colorectal cancer, the general issues identified above are similar to considerations encountered in the design of genetic studies of other common conditions such as other cancers, diabetes, hypertension and many other chronic diseases that arise from both environmental and genetic causes.

#### Allele Frequency and Penetrance

Most of the models we studied include a two-locus noninteracting model for which neither locus had been molecularly determined and only one was linked to the markers under consideration. The unlinked locus varied in allele frequency and penetrance. In some simulations the unlinked locus had no effect on disease, but in others the penetrance and/or allele frequency were assumed to be greater than that of the linked locus. The two loci contributed multiplicatively in a logistic model for penetrance of the form

logit Pr(*Y *= 1|*G*_1_, *G*_2_) = α + β_1_Dom(*G*_1_) + β_2_Dom(*G*_2_)

where Dom(*G*) indicates a dominant coding of the genotype, i.e. presence of one or two copies of the deleterious allele identically increases the risk of the disease. We also simulated some situations in which only one autosomal dominant predisposition syndrome was present in the population.

In the U.S. population, lifetime risks for colorectal cancer are approximately 2.5–5 percent [45]. In the context of a high-risk cancer clinic, however, patients are frequently seen at younger ages, and hence the background risk of colorectal cancer among sibships ascertained through high-risk clinics is considerably less than 5%. We set the natural log of the background rate of colorectal cancer in our simulations to α = -4, so that the overall population prevalence of disease was approximately 2%. Objective estimates of allele frequencies and relative risks for unknown mutations are not available, so we chose values that were consistent both with frequencies of known mutations [46,47] and with population attributable risk estimates for familial colorectal cancer [24,48]. As an example, with an allele frequency of *q*_1 _= 0.003 and a log relative risk of β_1 _= 3 (relative risk = 20.1), *q*_2 _= 0.001 and β_2 _= 4.6 (relative risk = 99.5), the resulting penetrances are 0.018 for carriers of neither mutation, 0.268 for carriers of a mutation in the linked gene, 0.646 for carriers of a mutation in the unlinked gene, and 0.973 for carriers of both mutations. The linked locus accounts for approximately 7.2% of colorectal cancers and the unlinked locus accounts for approximately 6.0% of colorectal cancers. Table 1 lists the parameters that we used in combinatios for the simulations, along with the values we assigned in various simulations.

**Table 1:** Parameters employed in simulations

**Parameter**	**Simulated Values**	
Families	500	
Affected Sibs Total	2, 3	
Affected Sibs Genotyped	1, 2, 3	
Unaffected Sibs Total	0, 1, 2, 3	
Unaffected Sibs Genotyped	0, 1, 2, 3	
Parents Genotyped	0, 1, 2	
Offspring Proxies	0, 1, 2	
Screen for Unlinked Syndrome	No, Yes	
Population Disease Prevalence	0.02, 0.05	
Mode of Inheritance	Autosomal Dominant	
Relative Risk to Carriers	10, 20	
Number of Disease Loci	0, 1, 2	
Disease Allele Frequency	0.003, 0.005, 0.01	
Marker Spacing (cM)	5.0, 10.0	
Admixture Models:	Population 1 (80%)	Population 2 (20%)
Marker Allele Frequencies	[0.25, 0.25, 0.25, 0.25]	[0.1, 0.2, 0.3, 0.4]
Disease Allele Frequencies	[0.00325, 0.0035]	[0.002, 0.001]

Parameters and parameter values employed in the simulations

### Sampling Plans

The primary concern in the design of CFRCCS-CGN colon cancer study is to maximize the relative efficiency per genotype, i.e., to find the combination of family structures and numbers of families that will produce the greatest power for a given number of genotypings. While the number of ASPs available for study will affect the power of the study, at the time when we were designing the study, approximately 425 ASPs (plus about 400 other relative pairs) were already available from the combined CFRCCS resources, and samples were already available from many of the potential subjects. An additional 500+ sib pairs were anticipated from the CGN sites. Thus, the primary constraint was the total cost of genotyping in a full genome scan, not the number of families. We therefore chose to concentrate on four main questions of efficiency: 1) whether (and how many) unaffected sibs should be genotyped, 2) how critical the absence of parental genotypes is to the efficiency of the analysis; 3) the relative loss in efficiency of genotyping spouses and offspring of a deceased affected sib, and 4) how different strategies for addressing genetic heterogeneity might affect these efficiency comparisons.

Although our primary interest was in the relative efficiency per genotype, we also wished to observe the effects on overall power and sample size requirements. We were particularly interested in the question of whether the total number of families that might be available for study in this large collaborative project would be adequate for the detection of linkage given a set of realistic assumptions concerning the genetics of colorectal cancer susceptibility.

### Linkage Tests for Concordant and Discordant Sib Pairs

Model-free linkage analysis is usually based on a comparison of the observed number of alleles at a marker locus shared *identical by descent *(IBD, i.e., derived from a common ancestor) between pairs of relatives with given phenotypes with that expected simply on the basis of their relationships. However, IBD status can only be determined unambiguously when both parents are different heterozygotes. Since late-onset diseases such as colon cancer severely restrict the availability of parental genotypes, the first step is to estimate the IBD probabilities from the marker data on all available pedigree members, using statistical approaches such as those as implemented in such software as *GENEHUNTER *[49], *GENIBD *[50], *Simwalk 2 *[51], or *Merlin *[52]. The family structure to be analyzed can include parents, affected and unaffected sibs, as well as the spouse and offspring of an unavailable affected sib if they were sampled.

For the simulation studies reported here, we compared the power and efficiency of two tests: the classic "means test" for linkage [53], which compares the observed proportion of alleles shared IBD against the expected proportion of 0.5; and a modified means test which compares the observed proportion of alleles shared IBD among ASPs with the observed proportion shared IBD among discordant sib pairs. The latter test has been suggested to control for potential bias (as discussed below) as well as to improve power. For this analysis, we followed the general approach described by Guo and Elston [54] but with modifications. Since combinations of pairs of IBD values within a family are not independent [55], we constructed the test using the mean IBD sharing among pairs of each type (concordant affected and discordant) within each family. We allowed for the correlation in mean IBD values for the concordant affected and discordant sib pairs by constructing a paired t-test that allowed for the correlation in mean IBD values between the sets of concordant and discordant pairs. Specifically, letting *p*_*fjki *_denote the estimated probability that sib pair (*j, k*) in family *f *= 1,. . .,*F *shares *i *= 0,1,2 alleles *IBD*, we first computed the mean *p*_*cf *_of (*p*_*fjk*1_/2 + *p*_*fjk*2_) in all affected pairs in family *f *and the corresponding mean *p*_*df *_in discordant pairs in the same family. We then computed the means *p*_*c *_and *p*_*d *_and standard deviations *s*_*c *_and *s*_*d *_of these quantities across families, and conducted a standard one-sample *t*-test comparing *p*_*c *_to 1/2 and a two-sample t-test comparing *p*_*c *_to *p*_*d*_, where F is the total number of families in the sample:

*Z*_*c *_= √F (*P*_*c *_- 1/2)/*S*_*c*_

*Z*_*d *_= √F (*P*_*c *_- *P*_*d*_)/√(*S*_*c*_^2 ^+ *S*_*d*_^2^)

both of which are tested against a standard Normal distribution. Note *Z*_*d *_does not take account of the correlation in IBD status between the two types of pairs within a sibship, so we used a paired *t*-test to allow for this, i.e.,

*Z*_*p *_= √F (*P*_*cf *_- *P*_*df*_)/*S*_*w*_

where *s*_*w *_denotes the empirical standard deviation of *p*_*cf *_- *p*_*df *_across families.

### Simulation Methods

For a given family structure and choice of parameters (described below), we simulated phenotype and marker data as follows. First, we generated genotypes **G **at the two disease loci by sampling from their respective population distributions (commonly called gene dropping). We then retained the simulated genotype vector with probability Pr(**Y**|**G**), where **Y **= (1,...,1,0,...,0) denotes the phenotype vector of the sibship under the design being considered (with *D *affected (*Y*_*i *_= 1) and *U *unaffected (*Y*_*i *_= 0) members). This process was continued until the total sample size of *F *families was ascertained. For each family structure, marker data were then simulated conditional on the genotypes at the linked locus. We assumed each marker locus had four alleles and that it was linked to the disease locus with two different recombination values of 5 and 10 centiMorgans between the disease and marker loci.

Regardless of the number of markers, marker alleles were simulated by dropping, for each parent, the parental allele corresponding to the chromosome of origin of the allele inherited at marker *l *- 1 with probability 1 - *θ*_*l*_, or the opposite chromosome with probability *θ*_*l*_, for marker loci *l = 1 ... L*, *l = 0 *at the disease locus, where *θ*_*l *_is the probability of recombination between loci *l *- 1 and *l*. All marker alleles were independent of the second (unlinked) disease locus, if one was employed in a given simulation.

We computed IBD probabilities for each marker locus by enumerating all possible alleles for each untyped founder and all possible segregation indicators for all nonfounders that are consistent with the observed marker data. We then accumulated the probabilities *p*_*fjki *_of each of these configurations into an array [56]. These probabilities depend upon the population marker allele frequencies, which we estimated in each replicate by a simple counting procedure from all the subjects available for study [57]. We did not adjust for correlations among family members as the estimates of allele frequencies so obtained are consistent [57] and are accurate for the large number of families we are here evaluating in each simulation.

In order to compare the test size of each linkage test we employed, we carried out 10,000 replicate simulations of *F *= 100 families under the null hypothesis of no linkage between the marker and either disease locus. For these analyses, we simulated families of two affected sibs, both genotyped, and two unaffected sibs, both genotyped.

We also considered the behavior of multipoint variants of the *Z*_*c *_and *Z*_*p *_statistics under the null. To calculate these, we initially simulated six markers with four alleles each spaced at 10 cM intervals, and obtained multipoint estimates of pairwise IBD sharing at 20 cM from the origin using the program Merlin [58], then calculated *Z*_*mc *_and *Z*_*mp *_using the formulas given above. The disease genes were unlinked to the six markers. We then repeated the simulations after reducing the marker distance by half. We did not go below 5 cM.

For each non-null simulation, we generated 20 replicate simulations of *F *= 500 families each. As a measure of cost efficiency per genotype, we divided the average *Z*_*c*_^2 ^(or *Z*_*p*_^2^) scores across the 20 replicates by the number of genotypes required, and reported the ratio (times 100) of this value to that based on *Z*_*c*_^2 ^for families consisting only of two sampled affected sibs. We call this the asymptotic relative efficiency (ARE). We chose to generate 20 replicate observations for any given combination of parameter values because we wished to simulate a wide variety of non-null conditions, while producing an adequate number of replicate observations to obtain reliable estimates of noncentrality parameters.

The number of families *F *required for a desired level of power sample was calculated in the standard way [59] by solving

$$\frac{Z_{\alpha }+Z_{1-\beta }}{\sqrt{F}}=\frac{Z_{sim}}{\sqrt{F_{sim}}}$$

for *F*, where *Z*_*α *_and *Z*_1-*β *_are the quantiles of the standard normal distribution for selected values of α and β, and *Z*_*sim *_is the sample mean of the Z-scores (*Z*_*c *_or *Z*_*p*_) based on the sample size *F*_*sim *_of the simulation run.

### Comparison of Sampling Strategies

The relative efficiency and absolute power comparisons were evaluated over a range of assumptions about disease allele frequency, penetrance, and heterogeneity. Summaries are provided for the relative efficiency from the non-null simulations of each combination of parameters and sample sizes required to attain 80% power. Our simulations involved the manipulation of 16 parameters, with over 725,000 possible combinations arising just among the limited set of settings we chose for each parameter. In practice, we were able to simulate 20 replicates for 332 actual parameter combinations. In order to assess the impact of each variable parameter setting on the power and efficiency of each study design, and to facilitate generalization of our results to other similar situations that we did not specifically simulate, we constructed linear regression models of the square root of the per-family contribution to the noncentrality parameter (*Z*_*sim*_/$\sqrt{F_{sim}}$) and ARE estimates resulting from each simulation as a function of the simulation parameters. Residuals from the models were approximately normally distributed, and scale-location plots did not suggest the data were heteroscedastic. We evaluated the fit of alternative regression models with quadratic, inverse, or logarithmic transformations of both the predictor and outcome variables; none fit the observed data better than a simple linear multiple regression model. The proportion of variance in *Z*_*sim*_$\sqrt{F_{sim}}$ explained by the linear regression (*R*^2^) ranged from 70–75%. The open source statistical package R [60] was used to perform all regression analyses.

In order to facilitate interpretation, the genetic effects (baseline disease prevalence, allele frequencies, and relative risks of the linked and unlinked loci) were collapsed into "attributable risks" (AR) for the linked and unlinked loci respectively. The attributable risk is the proportion of the total disease risk attributable to variation at each genetic locus. The fit of linear regression models employing the two AR values is as good as those employing the five original terms to fit the data, using the Akaike Information Criterion (not shown; [61]).

## Results

### Test Size

In Tables 2 and 3 we compare the performance of each linkage statistic under the null hypothesis of no linkage to its asymptotic expectation. The values in Tables 2 and 3 were obtained for 10,000 replicate observations of a design with two affected sibs (both sampled), and two unaffected sibs (both sampled), with no parents or offspring sampled. The observed mean and standard deviation of each statistic is reported, along with the probability of Type I error for assumed one-tailed test sizes ranging from 0.1 to 0.0005. Overall, the concordant test, *Z*_*c*_, and the paired test, *Z*_*p*_, most closely matched the nominal test size. Both the mean and standard deviation of *Z*_*c *_were close to theoretical expectations. The standard deviation of *Z*_*p *_was only slightly larger, and the mean was slightly less than zero (0.05 > p > 0.01). The standard deviation of *Z*_*d *_was about 9% larger than expected, so the significance of tests based on this statistic will be overstated unless adjustments are made. We therefore omit *Z*_*d *_from further consideration.

**Table 2:** Simulation results

	**Statistic**				
**One-Tailed p**	**Z**_c_^a^	**Z**_d_^b^	**Z**_p_^c^	**Z**_mc_^d^	**Z**_mp_^e^

**0.1**	0.0987	0.1169	0.0966	0.0663	0.0971
**0.05**	0.0505	0.0637	0.0451	0.0317	0.0473
**0.01**	0.0107	0.0170	0.0094	0.0028	0.0039
**0.005**	0.0053	0.0097	0.0048	0.0003	0.0010
**0.001**	0.0013	0.0022	0.0008	0.0001	0.0006
**0.0005**	0.0010	0.0013	0.0004	0.0001	0.0000
**Mean**	-0.005	-0.004	-0.0175	-0.246	-0.036
**SD**	1.002	1.087	1.004	1.015	1.016
**p-value**^f^	0.309	0.356	0.041	< 0.0001	0.0002

Observed versus expected distributions of linkage test statistics, based on 10,000 simulations of 100 families with the following configuration: 2 affected sibs, both sampled, and 2 unaffected sibs, both sampled, disease locus unlinked to markers (null model).

^a^mean concordant test; ^b^mean discordant (unpaired) test; ^c^mean paired test; ^d^mean multipoint concordant test (10 cM spacing between markers); ^e^mean multipoint paired test; ^f^p-value of test that mean does not equal 0;

**Table 3:** Simulation results under varied conditions of heterogeneity

	**Heterogeneity Model**					
	**Disease Allele Only**^g^		**Markers Only**^h^		**Disease and Markers**^i^	

**One-Tailed p**	**Z**_c_	**Z**_p_	**Z**_c_	**Z**_p_	**Z**_c_	**Z**_p_

**0.1**	0.0966	0.0939	0.1153	0.1022	0.1038	0.0951
**0.05**	0.0471	0.0426	0.0626	0.0515	0.0527	0.0442
**0.01**	0.0087	0.0084	0.0139	0.0096	0.0113	0.0088
**0.005**	0.0043	0.0034	0.0078	0.0044	0.0055	0.0034
**0.001**	0.0011	0.0006	0.0015	0.0005	0.0014	0.0008
**0.0005**	0.0009	0.0003	0.0006	0.0004	0.0006	0.0003
**Mean**	-0.015	-0.027	0.059	-0.007	0.041	-0.026
**SD**	1.001	1.007	1.019	1.017	1.004	1.008
**p-value**^f^	0.067	0.0037	< 0.0001	0.246	< 0.0001	0.0049

Observed versus expected distributions of linkage test statistics under varying conditions of disease allele and/or population heterogeneity in marker allele frequencies, based on 10,000 simulations of 100 families with the following configuration: 2 affected sibs, both sampled, and 2 unaffected sibs, both sampled, disease locus unlinked to markers (null model).

^e^mean multipoint paired test; ^f^p-value of test that mean does not equal 0; ^g^two populations modeled in an 80%/20% mixture: 1) marker allele frequencies for all markers [0.25,0.25,0.25,0.25], unlinked disease allele 0.0035; 2) marker alleles [0.25,0.25,0.25,0.25], unlinked disease allele 0.001; ^h^two populations modeled in an 80%/20% mixture: 1) marker allele frequencies for all markers [0.25,0.25,0.25,0.25], unlinked disease allele 0.003; 2) marker alleles [0.4,0.3,0.2,0.1], unlinked disease allele 0.003; ^i^two populations modeled in an 80%/20% mixture: 1) marker allele frequencies for all markers [0.25,0.25,0.25,0.25], unlinked disease allele 0.0035; 2) marker alleles [0.4,0.3,0.2,0.1], unlinked disease allele 0.001.

The mean multipoint estimate of allele sharing was significantly below 0.5 for concordant pairs in these null simulations, leading to a conservative bias in *Z*_*mc*_. *Z*_*mp *_is also conservatively biased, but much less so, because the statistic compares estimated sharing between concordant pairs with estimated sharing between discordant pairs, which is also estimated to be significantly less than 0.5 by the multipoint algorithm. Schork and Greenwood, Cordell, and others [62,63] recently demonstrated the bias toward the null that occurs when using an NPL statistic with an incompletely informative marker unless an adjustment is made for incomplete marker informativity. Since marker informativity decreases with increasing marker intervals, the increasing conservativism of the *Z*_*mc *_with increasing marker intervals is not surprising.

When we ran null simulations in the presence of heterogeneity of marker alleles, disease alleles, or both, we observed that *Z*_*c *_and *Z*_*p *_behaved differently depending on the heterogeneity model we simulated. Although *Z*_*p *_was conservatively biased when disease allele frequencies differed between populations, regardless of whether marker allele frequencies differed, its small conservative bias was not significantly different from that observed in the homogeneous population of Table 2. In contrast, *Z*_*c *_exhibited an anticonservative bias when marker allele frequencies differed between populations, regardless of whether disease allele frequencies differed.

### Alternative Model Simulation Results

A subset of results from alternative model simulations in which there were linkages are given in Table 4, based on 20 replicate simulations for each combination of parameters. The numbers given represent mean Z statistics, their ARE values, and sample sizes required to attain 80% power. The results displayed were chosen to highlight the effect of sampling (and genotyping) varying the numbers of unaffected sibs and/or parents from families with two or three affected sibs and two unaffected sibs. Full results are available online [64]. On average, the concordant test was always more efficient on a per-genotype basis than the paired test, although the power of the paired test was usually greater when the number of unaffected sibs tested was > 2.

**Table 4:** Alternative Model Simulation

***Model A – no heterogeneity:***										
	**Affected Sibs**		**Unaffected Sibs**							
**Parents**	**Total**	**Sampled**	**Total**	**Sampled**	**Z**_c_	**Z**_p_	**ARE(Z**_c_**)**	**ARE(Z**_p_**)**	**Nc(80%)**	**Np(80%)**
0	2	2	2	0	3.19		100		304	
0	2	2	2	1	2.70	2.42	72	38	424	528
0	2	2	2	2	3.03	3.17	90	49	337	308
0	3	2	2	0	6.09		364		83	
0	3	2	2	1	6.46	6.00	410	236	74	86
0	3	2	2	2	6.12	6.47	368	206	83	74
2	2	2	2		2.88		41		373	
2	2	2	2	1	3.17	2.69	49	28	308	427
2	2	2	2	2	3.17	3.33	49	36	308	279

***Model B – less common, equally penetrant locus:***										

0	2	2	2	0	2.37		100		550	
0	2	2	2	1	3.04	2.88	165	98	334	373
0	2	2	2	2	2.73	2.81	133	70	415	391
0	3	2	2	0	4.66		387		142	
0	3	2	2	1	5.29	5.11	498	310	110	118
0	3	2	2	2	4.86	5.18	421	239	131	115
2	2	2	2	0	2.73		66		415	
2	2	2	2	1	2.46	2.22	54	35	511	627
2	2	2	2	2	2.92	2.95	76	52	363	355

***Model C – equally common, equally penetrant locus:***										

0	2	2	2	0	2.38		100		546	
0	2	2	2	1	1.93	1.95	66	45	830	813
0	2	2	2	2	2.33	2.36	96	49	569	555
0	3	2	2	0	3.45		210		260	
0	3	2	2	1	3.47	3.33	213	131	257	279
0	3	2	2	2	3.48	3.84	214	130	255	210
2	2	2	2	0	2.26		45		605	
2	2	2	2	1	2.56	2.44	58	42	472	519
2	2	2	2	2	2.22	2.14	44	27	627	675

Mean Z score, asymptotic relative efficiency per genotype (ARE–see text), and sample size required to attain 80% power for two-point linkage (θ = 0.05) for predisposition to colorectal cancer with a baseline risk of 0.018, a relative risk to carriers of 20, allele frequency of 0.003, with a lifetime penetrance of 0.27, under 3 different heterogeneity models: A) no heterogeneity; B) an equally penetrant, less common (allele frequency 0.001) unlinked predisposing locus; C) an equally penetrant, equally common unlinked predisposing locus. All results based on 500 simulated families and 20 replicate simulations per parameter set.

Z_c _mean concordant test; Z_p _mean paired test; ARE(Z_c_) asymptotic relative efficiency concordant test; ARE(Z_p_) asymptotic relative efficiency paired test; Nc(80%) number of families required for 80% power using concordant test; Np(80%) number of families required for 80% power using paired test.

### Regression Models

Table 5 shows the effect of various design and population parameters on the noncentrality parameter for both the concordant-only test (*Z*_*c*_/$\sqrt{F}$) and the paired test (*Z*_*p*_/$\sqrt{F}$). The only change in parameterization of the model for the paired test vis-a-vis the concordant test involved substituting the number of typed unaffected sibs for the total number of unaffected sibs. The AR for the linked locus had the largest positive effect on power of the concordant test, followed closely by the negative effect on power of increasing AR at the unlinked locus and increasing recombination fraction between the marker and trait loci. Increasing the number of affected sibs per family and increasing the number of affected sibs genotyped per family both substantially improved power. The number of unaffected sibs present in sampled families had little or no effect on power. Increased power was also observed for increasing numbers of available parents, the size of the effect on *Z*_*c*_/$\sqrt{F}$ being about 15% of the effect of increasing numbers of affected sibs. Substituting spouse and offspring for an unavailable affected sib reduced power substantially, but genotyping a second child recovered much of that lost power.

**Table 5:** Effect of design and population parameters or non-centrality parameters

	Simulated Range	Z_*c*_/√F		Z_*p*_/√F	
		
		Estimate	SE^a^	Estimate	SE
(Intercept)		-0.3392	0.0064	-0.2941	0.0085
Recombination Fraction	0.025–0.25	-0.5783	0.0247	-0.5757	0.0228
Total Affected Sibs	2–3	0.0948	0.0020	0.1088	0.0026
Affected Sibs Typed	2–3	0.1111	0.0022	0.0670	0.0029
Total Unaffected Sibs^b^	0–3	0.0003	0.0011	0.0043	0.0021
Parents Typed	0–2	0.0156	0.0009	0.0099	0.0012
Spouse-Offspring Proxy	0–1^c^	-0.0222	0.0028	-0.0179	0.0041
Spouse-2 Offspring Proxy	0–1 ^c^	-0.0050	0.0027	-0.0050	0.0038
AR Linked Locus	0.025–0.12	1.5785	0.0360	1.5154	0.0402
AR Unlinked Locus	0.0–0.12	-0.9867	0.0252	-0.7830	0.0295
Screen for Unlinked (AR1 > AR2)	0–1 ^c^	-0.0016	0.0057	0.0054	0.0053
Screen for Unlinked (AR1 ≤ AR2)	0–1 ^c^	0.0666	0.0042	0.0661	0.0040

Multiple linear regression of *Z*/$\sqrt{F}$ on simulation parameters.

^a^Standard error of Estimate; ^b^Number available for *Z*_*c*_, number genotyped for *Z*_*p*_; ^c^Indicator variable.

In general, the effects on power of changes to the genetic and design parameters were very similar for the discordant test. Increasing the number of unaffected sibs typed did yield a small increase in power, but increasing the number of affected sibs, parents or offspring proxies increased power by a larger amount, even with the discordant pair test.

The effect of experimental screening for the unlinked syndrome varied substantially depending on the relative magnitude of the effects of the unlinked syndrome: if the unlinked syndrome had an attributable risk greater than or equal to that of the linked syndrome, experimental screening resulted in a large increase in power. Otherwise, the effect of experimental screening was negligible.

Power for a given sample size *F *and test size α with a specified combination of design and genetic parameters can be derived from the regression models in Table 5 by first obtaining an estimate of the noncentrality parameter *Z*/$\sqrt{F}$ from the appropriate column of Table 4, multiplying by $\sqrt{F}$ to obtain $\hat{z}$, and then solving for *β *in the equation below:

$$\beta =\Phi (Z_{\alpha }-\hat{z}),$$

where *β *is the probability of Type II error (hence power is 1-*β*) and Φ(*z*) is the cumulative standard normal distribution. A script for computing the predicted power for any particular combination of these parameters, using the statistical languages S-plus or R is also available from the website [65].

**Figure 1:**
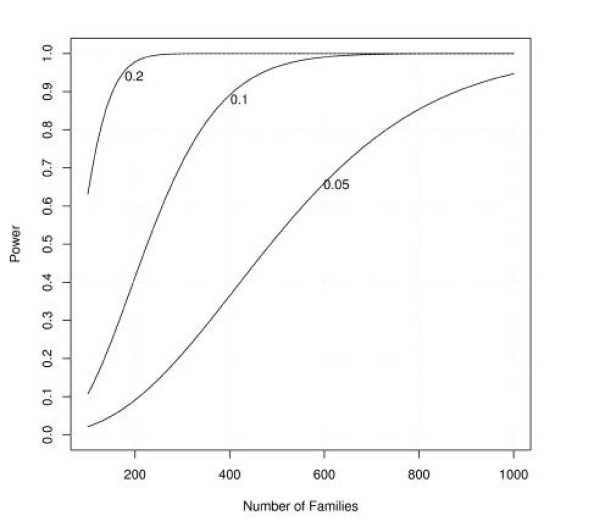
Effect of sample size and varying attributable risk at the linked locus on estimated power.

**Figure 2:**
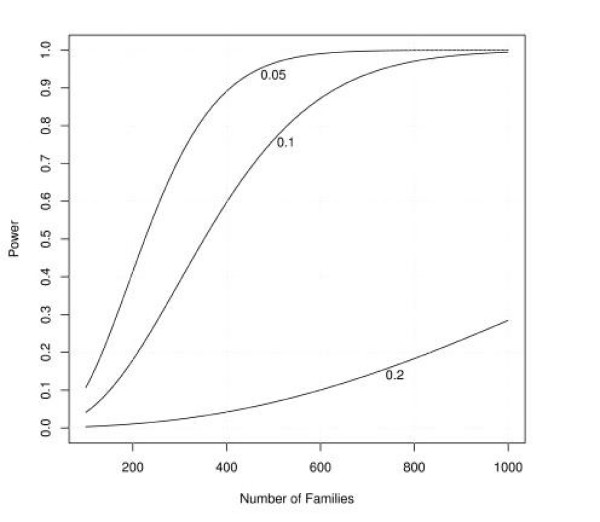
Effect of sample size and varying attributable risk at the unlinked locus on estimated power.

**Figure 3:**
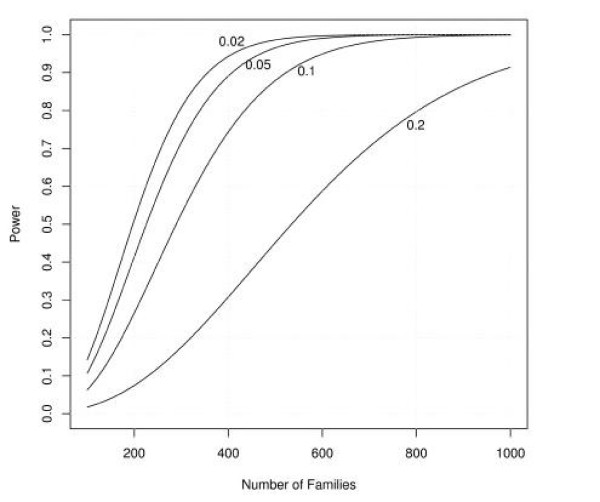
Effect of sample size and varying recombination fraction on estimated power.

**Figure 4:**
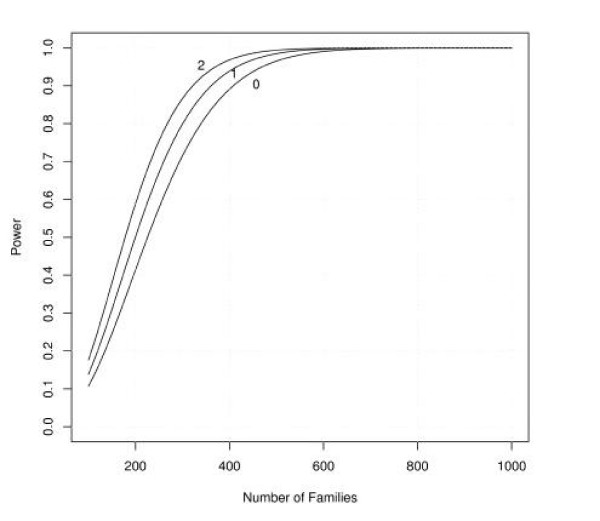
Effect of sample size and varying number of typed parents on estimated power.

**Figure 5:**
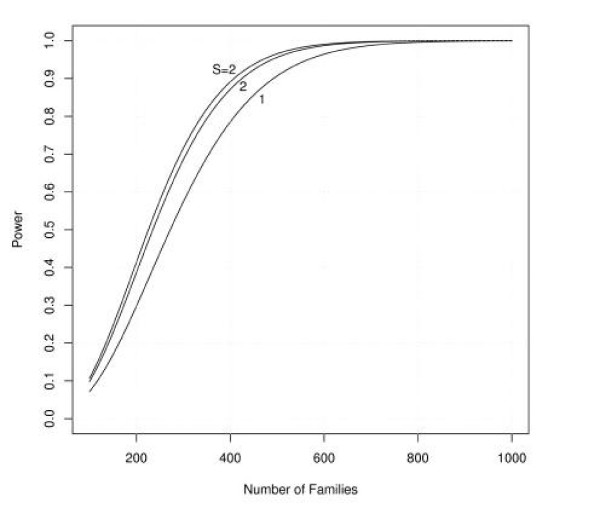
**Effect of varying number of offspring proxies on power for families of two affected siblings**. Effect of sample size and varying numbers of offspring proxies (1 or 2) on estimated power, families with two affected sibs, with only one available for genotyping (1 or 2), compared with families with both affected sibs available for genotyping (S = 2).

**Figure 6:**
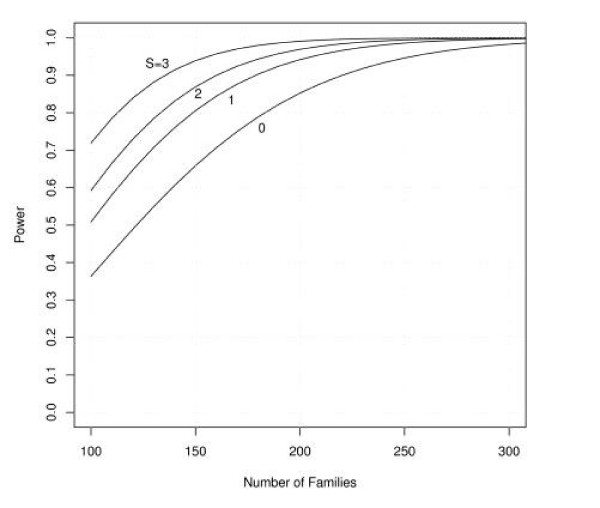
**Effect of varying number of offspring proxies on power for families of three affected siblings**. Effect of sample size and varying numbers of offspring proxies (O) on estimated power, families with three affected sibs, with only two available for genotyping (O = 0, 1, or 2), compared with families with all three affected sibs available for genotyping (S = 3).

Figures 1, 2, 3, 4, 5, 6 show estimated power of the *Z*_*c *_test for varying sample sizes given several values of AR for the linked and unlinked loci, recombination fraction (_*θ*_), number of parents genotyped, and number of offspring proxies. Except as noted, the power estimates are based on the following settings of the genetic and design parameters: two total affected sibs, both sampled, two unaffected sibs (not sampled), no parents or offspring proxies, AR1 (linked locus) = 0.1, AR2 (unlinked locus) = 0.05, *θ *= 0.05, and no screening to exclude unlinked families.

### Relative Efficiency of Alternative Designs

Because the power of the discordant test was rarely much greater than that of the concordant test, and the concordant test requires genotyping of only the affected sibs, the ARE of the concordant test was always greater on average than that of the discordant test. Nevertheless, we were interested in specific combinations of design and genetic parameters that might have resulted in better relative efficiency for the discordant test. Overall, the *ARE *for the discordant test was larger than that for the concordant test in 13% of the replications. We used a logistic regression model with the same parameters as those in Tables 2, 3 and 4 to predict the probability that *Z*_*p *_would outperform *Z*_*c *_in terms of relative efficiency (not shown). Only under circumstances that would result in very low power, such as attributable risks for the unlinked locus more than twice as large as attributable risks for the linked locus, or recombination fractions > 0.1, was *Z*_*p *_predicted to outperform *Z*_*c *_in our simulated sample of 500 families.

In principle, including genotype data from unaffected siblings can improve the ability to estimate haplotypes and thereby improve power when multipoint data are studied. Table 6 compares estimates of *Z*_*c *_and *Z*_*p *_for a selected set of simulations with multipoint *Z*_*m *_scores for the same families as calculated by the computer program Merlin [58]. In these simulations, when markers were spaced no closer than 10 cM from one another, as is usually the case in an initial genome scan, we observed no improvement in power with the concordant multipoint statistic, but an improvement in power with the discordant test. When markers were spaced at 5 cM intervals, however, the power of both multipoint statistics to detect linkage exceeded that of both *Z*_*c *_and *Z*_*p*_. The conservative bias of *Z*_*mc *_noted in Tables 2 and 3 appears to decrease as marker intervals become smaller. To test this, we conducted additional simulations under the null hypothesis as described above for Tables 2 and 3, with 1000 repetitions and markers spaced at 5, 10, and 20 cM. The magnitude of the conservative bias in *Z*_*mc *_decreases consistently (mean *Z*_*mc *_= -0.58 at 20 cM, -0.23 at 10 cM, and 0.06 at 5 cM) as marker intervals contract, becoming significantly anti-conservative at 5 cM.

**Table 6:** Comparison of two-point vs. multipoint linkage scores under various non-null simulation conditions.

**Simulation Model**	**Marker Interval**	***Z***_c_	***Z***_p_	***Z***_mc_	***Z***_mp_
No Heterogeneity ^a^	5 cM	3.26	3.24	3.62	3.60
	10 cM	2.94	2.91	2.85	3.36
	20 cM	2.36	2.30	2.14	2.52
Locus Heterogeneity^b^	5 cM	3.10	3.08	3.75	3.53
	10 cM	2.76	2.70	2.72	3.13
	20 cM	2.05	2.01	1.91	2.23
Marker Heterogeneity – Mild^c^	5 cM	2.80	2.72	3.57	3.24
	10 cM	2.62	2.54	2.59	2.99
	20 cM	2.22	2.13	2.14	2.35
Marker Heterogeneity- Severe^d^	5 cM	2.10	1.95	2.61	2.35
	10 cM	1.70	1.56	1.49	1.86
	20 cM	1.42	1.27	1.18	1.33

All simulations employed 20 replicates of 500 families with 2 affected and 2 unaffected sibs, all sibs typed, no parents or children typed.

^a ^Baseline risk of 0.018, a relative risk to carriers of 20, allele frequency of 0.003, with a lifetime penetrance of 0.27, no genetic heterogeneity.

^b ^Baseline risk of 0.018, a relative risk to carriers of 20, allele frequency of 0.003, with a lifetime penetrance of 0.27, and a less common (allele frequency 0.001), equally penetrant unlinked predisposition syndrome.

^c ^Two populations modeled in an 80%/20% mixture: 1) marker allele frequencies for all markers [0.25,0.25,0.25,0.25], disease allele 0.00325; 2) marker alleles [0.4,0.3,0.2,0.1], disease allele 0.002.

^d ^Two populations modeled in an 80%/20% mixture: 1) marker allele frequencies for all markers [0.25,0.25,0.25,0.25], disease allele 0.0035; 2) marker alleles [0.4,0.3,0.2,0.1], disease allele 0.001.

Replacement of a missing affected individual by a spouse and offspring is not generally as efficient per genotype as if that individual had been available, although the efficiency improves with each additional offspring included. Figure 5 shows the effect of replacing one member of an ASP with one or two offspring and the spouse. Figure 6 shows the effect of replacing one member of an affected sib trio with one or two offspring and the spouse. Because families with three affected sibs are more likely to be segregating a disease-predisposing allele than families with two affected sibs, each family is more informative for linkage, even if DNA is available for only two sibs. Substituting the spouse and one or more offspring for the missing sib adds additional power, although at the cost of additional genotyping.

## Discussion

In studying a disease like colon cancer with both incomplete penetrance and a high incidence of sporadics, prior studies have shown that to be efficient, families must include two or more individuals with the same disease from whom samples can be obtained. The practical experience of many investigators suggests that once an initial entree is gained into a family, the chances of enrolling other members are quite high. Close relatives are often very motivated to participate in research into a disease that has afflicted their family, especially when the commitment is minimal, involving only a blood specimen and a signed consent, which are all that are required for a linkage study of diseases that have an easily established phenotype, like cancer. In our simulations, we considered inclusion of unaffected sibs and the use of spouse and offspring to replace missing affected sibs. Including unaffected siblings in the design of a study can improve the power to detect linkage for highly-penetrant diseases [53,66]. However, our simulation studies demonstrate that for the model-free linkage tests we studied, for low penetrance loci, and with no genotyping error, little increase in power can be gained by including unaffected siblings in the design of a sib-pair study of a late onset, complex disease such as colorectal cancer. The inclusion of unaffected siblings can be useful, however, in reducing anti-conservative biases arising from heterogeneity or misspecification of marker allele frequencies or genotyping errors, and conservative biases arising if multipoint statistics are used with an insufficiently dense marker set. Table 2 shows that, with markers spaced at 10 cM intervals, *Z*_*mc *_is conservatively biased, while exhibiting an anticonservative bias in Table 3 under conditions of heterogeneity in marker allele frequencies. In Table 6, *Z*_*mp *_outperforms with markers spaced at 10 and 20 cM. At 5 cM, *Z*_*mc *_becomes anticonservatively biased, as noted above. It may be useful to perform simulations to assess the characteristics of the tests when the marker spacing decreases, as would be possible with the newer SNP array-based platforms. Further, since genome-wide-association studies do not utilize the full power of the sibpair design, future research should be aimed at assessing the optimal setting for relying on the sibpair design, as it is premature to move completely away from this study design [15].

If a secondary goal of a study is to estimate the penetrance, then unaffected relatives should be included in the design of the study; also unaffected siblings could help in evaluating the impact of environmental factors upon disease risk. Genotyping errors for microsatellites scans are currently very low but are hard to assess without the availability of parents or siblings. Our findings parallel those of Holmans et al. [67], who suggested that genotyping affected individuals only is efficient.

Unaffected siblings can be used for studies that take advantage of linkage disequilibrium to identify associations. Colorectal cancer and other late onset diseases with incompletely penetrant susceptibility alleles complicate the identification of unaffected individuals for association studies, because the probability that a person who does not currently exhibit the disease will become affected sometime in the future can be relatively large. In association studies using families, the oldest unaffected sibling is the most informative because s/he is more likely to have attained the age at which all the cases in the family were diagnosed (although Kraft and Thomas [68,69] have shown how younger siblings can also be used).

The inclusion of unaffected sibs is also advantageous with respect to the common practice of genotyping in batches, particularly by nuclear family. A correlation may be induced artificially if the genotyping errors are resolved within families by requiring Mendelian segregation of alleles, which can lead to false positive evidence for linkage, unless the unaffected relatives are used for comparison. Such excess evidence for linkage tends to accrue from the families with available parents because these pedigrees are the mostly likely to yield non-Mendelian segregation due to genotyping errors, which have to be resolved in turn [70,71]. Good laboratory practice requires that at least some samples receive duplicate genotyping so that it would be possible to identify problematic markers showing excess genotyping error rates. Meiotic drive exists in some mammals and if present in humans could conceivably increase the expected IBD sharing at a locus above 0.5. If meiotic drive is present in humans then multiple studies of many different diseases should show this effect [72].

One sampling option that we have shown can be viable includes sibships in which an affected member is dead but the spouse and offspring of the deceased are available, thus effectively increasing the number of pairs and yielded a total information content per family approaching other designs based on affected sibs. In the context of a high-risk clinic, pursuing the family of a dead sib can be quite labor intensive compared with the cost of recruiting more sibships. However, in an established registry, the opposite may be true, so that extending the relatives to be sampled becomes a more cost-effective strategy. Such a scheme could be worthy of further consideration in situations where the prospects of recruiting additional sibships are limited. Using spouses and offspring as proxies for missing or deceased sibs is obviously less efficient than had the affected sibs themselves been available, but still can provide adequate linkage information and may be required if the disease is often lethal so that samples from affected sibs are hard to obtain. Even when affected siblings are unavailable for study, it is still possible to genotype blocks that may be available from residual tissues, such as lymph nodes, that were obtained for the purposes of staging. However, genetic analysis of blocks is more difficult than from blood samples because the DNA is partially degraded, which may preclude multiplex genotyping. Therefore, genotyping the relatives of the affected but unavailable cosib may be more efficient than genotyping residual tissues.

Available parents contributed somewhat to the efficiency obtained in our simulated linkage studies. There are other benefits of including parents when they are available, including greatly reduced sensitivity to allele frequency misspecification, substantial improvements to haplotype estimation, and identifying nonpaternities or other errors in family structure or in genotyping. There are some practical advantages to including both parents of each sibship, whenever possible. However, parents are likely to be available only for a small minority of the families because most inherited cancers have a relatively late onset, albeit typically earlier than for sporadic cases. For example, data from the high-risk families of the CFRCCS and the population-based Diet, Activity and Reproduction in Colon Cancer (DARCC) study [26] show that about 3–4% of the living ASPs have two parents alive while about 12% (DARCC) to 22% (CFRCCS) have only one. The sib pair protocol of the Eastern Cooperative Oncology Group (ECOG) for mapping the interactive susceptibility loci of four major malignancies includes the accrual of parents only when both are available. However, their rationale is quite different from that of allele-sharing methodology because the parental data are collected for a separate parallel analysis using the transmission disequilibrium test (TDT) [73].

The family structures that could be studied are not necessarily restricted to the sibs of a proband ascertained through a registry or clinic. Consideration should also be given to collecting the extended family members of the affected sibs. Familial cancer registries such as the CFRCCS and CGN routinely collect family history from all their participants. Recent reports indicate that study subjects are capable of accurately reporting cancer status among their close relatives, with accuracy varying by cancer site and degree of relationship [74]. Pedigrees that extend to affected aunts and uncles or grandparents of the affected sibs could be used in a confirmatory analysis for the source of the segregating disease allele by the method of Olson and Elston [75], without obtaining their blood specimens, by correlating the parent of origin of shared alleles with the side of the family of the affected aunt or uncle.

For rare autosomal dominant traits, Risch [76] showed that cousin pairs actually provide more information for detecting tight linkage than sib pairs. Narod and Amos ([77]) also showed through simulation the efficacy of incorporating cousins in a linkage study of breast cancer, which like colorectal cancer shows genetic heterogeneity, a likely high proportion of phenocopies, and environmental and life-style related risk factors. However, Risch also found that for recessive conditions or when the disease and marker loci were not tightly linked, a sib pair design was more effective than other designs. Since the mode of inheritance for as yet unidentified genetic factors remains unknown, the sibship design remains preferable to other family units since it provides power under all genetic models.

We estimated the numbers of families required to study colorectal cancer and similar diseases with reasonable power, if our assumptions about disease allele frequencies, mode of inheritance, and heterogeneity are not too far from the truth. Our simulation studies show that at least 500–1000 families are required to provide reasonable assurance of adequate power across a range of genetic models that are consistent with the observed familiality and population prevalence of colorectal cancer. Colon cancer risk is strongly associated with certain environmental factors, some of which might interact with the gene(s) being sought. Although 1000 pairs will most likely provide adequate power to identify genetic risk factors, the further delineation of gene-environment interactions may yet require larger sample sizes [78]. Our simulation study focused upon efficiency of various genotyping approaches, where we assumed that samples from individuals are already available. If samples are not available, then additional design constraints may become important. For instance, the collection of sib pairs affected by disease can be a slow process and trade-offs between efficiency in genotyping versus power from available samples may need to be evaluated.

Because the costs of drawing blood and extracting DNA are far smaller than those of genotyping the 400–1000 markers needed for a genome scan, and because the unaffected and affected siblings can contribute different information, we suggest collecting all available members of each sibship, but only genotyping affected sibs, parents, and any spouse/offspring proxies for an initial genome scan. DNA from the unaffected sibs may still be useful for fine mapping and association studies and for estimating the penetrance. The CFRCCS and DARCC databases indicate that about 90% of the living ASPs have at least one unaffected sib alive, with a mean of about 2.5. Since the optimal number of unaffected sibs to sample per sibship if more than one were available has not been resolved, an ad-hoc plan may be to obtain the blood specimens from all those who consent. If the genotyping resources were constrained (e.g. to at most two unaffecteds per sibship), an efficient protocol is to start with the oldest first-degree relative and to proceed downwards, i.e. include both parents if available, otherwise one parent and the oldest unaffected sib, etc.

Population heterogeneity and allele frequencies have an important impact on the study design. In the absence of parents, the marker allele frequencies in the study population have to be estimated as the basis for estimating the IBD probabilities, which requires an assumption of homogeneity of marker allele frequencies. Population heterogeneity arises when the allele frequencies vary across subgroups, because of demographic, geographical, or other factors. Table 3 shows that an incorrect assumption of population homogeneity so the assumption of a common allele frequency across the entire collection of families can bias affecteds-only linkage results, depending on the degree of admixture and the degree to which the allele frequencies vary among the subpopulations.

We observed moderate to substantial losses of power to detect linkage in the limited number of simulations we performed with population heterogeneity of this sort (see Table 6). The use of unaffected sibs provides one means of overcoming the problem of heterogeneity in marker allele frequencies, since both concordant and discordant pairs tend to be biased in a similar manner by any misspecification of allele frequencies. To fully exploit this advantage, however, a matched comparison is needed so that the analysis focuses on the within-family differences in IBD probabilities between the two types of pairs. This would overcome any problems of imbalance in the ethnic distribution of concordant and discordant pairs. While this can be accomplished by means of a simple paired *t*-test, like *Z*_*p*_, a more sophisticated GEE-2 approach is possible, modeling the entire vector of phenotypes in each family in relation to its IBD matrix [79]. This would have the advantage of combining information from both types of comparison in a matched fashion, so that all families, including those with no unaffected sibs, would contribute. The simulations summarized in Table 6 do not show any power advantage for either *Z*_*p *_or multipoint approaches under the simulated conditions of population admixture, although the anti-conservative bias in *Z*_*c *_under heterogeneity of marker allele frequencies may partially obscure a real power difference between the concordant and discordant tests. Population admixture presents a particularly insidious threat to the validity of tests for linkage if parents or unaffected siblings are not available. However, it might be possible to detect population substructure by checking for the departure from disequilibrium that it engenders.

We did not directly study the asymptotic relative efficiency of 10 cm versus 5 cm linkage scans. At the time we designed our study, the usual practice in genotyping facilities is to first apply a 10 cm map and then to follow up with denser mapping in linked regions. We generally anticipated that an initial 5 cM mapping strategy is not cost effective and that some version of grid tightening would ordinarly be employed [67]. Currently with the availability of more modern technology, a 10 cM scan is now considered rather sparse, and the 5 cM scan is considered a better standard, with fine mapping at 1 cM (although you still see published studies that use 10 cM scans; see [16-18]). Many studies now use SNP platforms, although it is not clear whether these are optimal for sibpair studies. When dense sets of SNPs are analyzed, there is much more potential for biases to appear if parental data are unavailable and linkage disequilibrium among the markers is not adequately modeled [80]. Such biases are reduced when an additional unaffected sibling or a parent is included in the analysis. Other than the need to address the potential for bias, the high marker informativity of microsatellites compared with SNPs would suggest that results from our simulation studies should shed light on optimal designs for studies of small families, whether microsatellites or SNPs are used for analysis.

Crude stratification by recruitment center is unlikely to be reliable enough for controlling population heterogeneity without additional adjustment. Stratification by ethnicity is generally more crucial than by geography, although both factors should ideally be incorporated. However, collection of ethnicity is not entirely trivial if it is to be worthwhile. Although various coding systems have been established by different agencies, no consensus has been established regarding the level of detail required on ethnicity data in genetic research, e.g. category distinctions, mixed-race marriages. Mandal *et al*. [81] have shown that marker allele frequencies are less important if there are four or more persons per family, especially when very polymorphic markers are used. When the parents of the sibpair have been genotyped, then there is no dependence on population marker allele frequencies and no source of bias in linkage analysis.

## Conclusion

We have shown that for a wide variety of situations relevant to sib-pair studies of colon cancer and similar late-onset complex diseases, standard concordant-pair allele-sharing statistics based on identity by descent probabilities are more efficient than similarly constructed discordant statistics in homogeneous populations, but may be biased away from the null in situations where subjects are chosen from multiple populations having differing marker allele frequencies or other sources of bias. Where heterogeneity in marker allele frequencies is anticipated because of underlying population admixture, linkage tests based on comparison of concordant vs. discordant allele sharing should be considered. Either of these approaches should allow studies of 500–1000 families containing an ASP to achieve reasonable power to detect linkage under conditions including genetic and population heterogeneity, unavailable parents, misspecified allele frequencies, and other afflictions of the sib-pair design. While collecting DNA samples from unaffected sibs can be useful for a wide variety of purposes–especially if multipoint methods are used–in many circumstances it should be unnecessary to genotype these samples for purposes of performing a genome scan. If adequate allele frequency data are available and microsatellites are being used, we suggest a single-point affecteds-only analysis for a initial scan, followed by a multipoint analysis of affected and unaffected members of all available sibships with additional markers around initial hits. For dense SNP mapping studies, analysis using individual SNPs will not be sufficiently informative and so multipoint analyses will be required, but unless the parents are all available, analyses should be restricted to markers that show low levels of linkage disequilibrium (the reference here would be to a paper by Bacanu SA. Multipoint linkage analysis for a very dense set of markers Genet Epidemiol. 2005 Nov;29(3):195–203.) or estimate haplotype frequencies (Abecasis and Wigginton, Handling marker-marker linkage disequilibrium: pedigree analysis with clustered markers. Am J Hum Genet. 2005 Nov;77(5):754–67. Epub 2005 Sep 20.) If the parents are available, even though we have shown that genotyping them is inefficient, an argument can be made for genotyping them because their inclusion ameliorates any potential effect of population admixture, greatly facilitates the identification of any genotyping errors and reduces the dependence upon correct specification of haplotype frequencies when multipoint analyses are conducted (can refer to Huang et al, 2004 Ignoring linkage disequilibrium among tightly linked markers induces false-positive evidence of linkage for affected sib pair analysis.

Am J Hum Genet. 2004 Dec;75(6):1106-12. Epub 2004 Oct 18.). The choice of whether or not to genotype unaffected individuals also would depend upon the feasibility and cost of cherry-picking for regional genotyping of regions showing evidence for linkage, but we have not studied either of these issues in this paper. Admixture is more likely to be of concern when studying populations that are known to have been founded by different ethnic groups or races such as Hispanics and African-Americans.

We have also observed that, if genetic testing is available for previously-identified syndromes, and if those syndromes are thought to account for a larger portion of the disease burden than the syndrome of interest, prior exclusion of families linked to the known syndrome can meaningfully increase the power of the study.

Finally we note that the colon sibpair study, for which this paper was written, was completed after accruing less than 100 families. In fact, most of the colon sibpair studies that have been published include fewer than 100 families, because recruitment is a major challenge. Although the results discussed here suggest numbers that may be challenging to accrue, the published literature has reported positive studies, suggesting that the assumptions used in our calculations may have been too conservative. In addition, linkage studies are meant to be performed in an iterative fashion so that the results from ongoing studies can be combined. Since our conclusions and recommendations arising from the simulations describe relative advantage of various designs, our results are of value in future study design.

## Competing interests

The authors declare that they have no competing interests.

## Authors' contributions

RAK took the lead in developing the results (performed simulations) and in writing the manuscript; CIA was a major participant in developing the results and preparing the manuscript; BYY drafted the manuscript and participated in manuscript preparation discussions, DMF coordinated the draft and development of the manuscript and participated in manuscript preparation discussions, DCT was a major participant in developing the results and preparing the manuscript. All authors read the final manuscript.

## Pre-publication history

The pre-publication history for this paper can be accessed here:


